# Plant–pollinator interactions over time: Pollen metabarcoding from bees in a historic collection

**DOI:** 10.1111/eva.12707

**Published:** 2018-11-13

**Authors:** Annemarie Gous, Dirk Z. H. Swanevelder, Connal D. Eardley, Sandi Willows‐Munro

**Affiliations:** ^1^ Biotechnology Platform Agricultural Research Council Pretoria South Africa; ^2^ School of Life Sciences University of KwaZulu‐Natal Scottsville, Pietermaritzburg South Africa; ^3^ College of Agriculture and Environmental Sciences University of South Africa Florida South Africa; ^4^ Plant Protection Research Institute Agricultural Research Institute Pretoria South Africa

**Keywords:** historic bee specimens, insect collection, ITS, *Megachile*, palynology, plant–pollinator interaction

## Abstract

Pollination is a key component in agricultural food production and ecosystem maintenance, with plant–pollinator interactions an important research theme in ecological and evolutionary studies. Natural history collections provide unique access to samples collected at different spatial and temporal scales. Identification of the plant origins of pollen trapped on the bodies of pollinators in these collections provides insight into historic plant communities and pollinators’ preferred floral taxa. In this study, pollen was sampled from *Megachile venusta* Smith bees from the National Collection of Insects, South Africa, spanning 93 years. Three barcode regions, the internal transcribed spacer 1 and 2 (ITS1 and ITS2) and ribulose‐1,5‐biphosphate carboxylase (*rbcL*), were sequenced from mixed pollen samples using a next‐generation sequencing approach (MiSeq, Illumina). Sequenced reads were compared to sequence reference databases that were generated by extracting sequence and taxonomic data from GenBank. ITS1 and ITS2 were amplified successfully across all (or most) samples, while *rbcL* performed inconsistently. Age of sample had no impact on sequencing success. Plant classification was more informative using ITS2 than ITS1 barcode data. This study also highlights the need for comprehensive reference databases as limited local plant sequence representation in reference databases resulted in higher‐level taxon classifications being more confidently interpreted. The results showed that small, insect‐carried pollen samples from historic bee specimens collected from as early as 1914 can be used to obtain pollen metabarcodes. DNA metabarcoding of mixed origin pollen samples provided a faster, more accurate method of determining pollen provenance, without the need for expert palynologists. The use of historic collections to sample pollen directly from pollinators provided additional value to these collections. Sampling pollen from historic collections can potentially provide the spatial and temporal scales for investigations into changes in plant community structure or pollinator floral choice in the face of global climate change.

## 1 INTRODUCTION

Our daily diet contains many plant products produced as a result of pollination, such as fruits, vegetables, nuts and seed‐derived commodities. This crucial ecosystem service not only ensures food on our tables, but also the diversification and maintenance of natural plant populations (Daily et al., 1997; Klein et al., 2007; Kremen et al., 2007). Studying the interaction between plants and their pollinators has traditionally been done by field‐based observation (Johnson, 1997; Wester, Stanway, & Pauw, 2009) and palynology (Dafni, 1992; Wilcock & Neiland, 2002). These methods are tedious and time‐consuming, and require experts in the fields of palynology and taxonomy to identify both the pollen and the pollinator. Similar pollen morphologies, especially from closely related taxa, further complicate plant identification by palynology (Hargreaves, Johnson, & Nol, 2004; Mullins & Emberlin, 1997; Williams & Kremen, 2007). These requirements have limited studies on plant–pollinator interactions for many pollinator genera, particularly in species‐rich regions where there is an abundance of both plants and pollinators.

Taxonomic activities in the areas of entomology and botany drive pollinator and palynology‐related work, but studies are often conducted independently from each other. Samples are often collected for taxonomic purposes, such as species identification, distribution pattern determination or identifying new introductions. Individual specimens are labelled with descriptive collection information, including collection date, location, collector and other relevant information before being stored in collection (Pennisi, 2000). Flower‐visiting animals housed within natural history collections may have pollen on their bodies. Although flower visitors were likely not collected with the aim of utilizing the pollen that was inadvertently collected along with the specimen, this pollen holds important information on the plants visited by the insect visitor, the identity of a possible pollinator and the plant community structure where the organism was collected. Additionally, a number of specimens from the same area, but from different temporal points, can be used to provide a chronological map of the area's plant and pollinator history. Historic collections may therefore provide a meaningful resource to investigate, not only pollinator–plant interactions over time, but also plant community change over time, providing important information on diversity and distribution.

DNA barcoding allows for identification and classification of organisms based on a short nucleotide sequence (Hebert, Cywinska, Ball, & deWaard, 2003). Ideal DNA barcodes have significant interspecific genetic variation and are flanked by conserved regions for universal primer binding to allow easy amplification across a wide range of taxa (Kress & Erickson, 2008). There is still debate on the optimal DNA barcode for plants (Dong et al., 2015; Ferri et al., 2015; Kress, García‐Robledo, Uriarte, & Erickson, 2015), but the ribulose‐1,5‐biphosphate carboxylase (*rbcL*) and maturase K (*matK*) chloroplast genes have been suggested as good candidate genes to target (CBOL Plant Working Group, 2009). Other chloroplast genes and regions have also been used successfully to barcode plants and pollen, including *trn*L (Kraaijeveld et al., 2015; Valentini, Miquel, & Taberlet, 2010), *rpoC1* and *trnH‐psbA* (CBOL Plant Working Group, 2009). Another well‐utilized marker is the internal transcribed spacer 2 (ITS2) region that is found between the 5.8S and 26S rRNA genes in plants (Chen et al., 2010; Yao et al., 2010). ITS2 has been used as the DNA barcode in recent pollen barcoding studies (Bell et al., 2017; Keller et al., 2015; Richardson, Lin, Quijia, et al., 2015; Sickel et al., 2015). ITS1 can be similarly useful at identifying plants to species level (Wang et al., 2015). Generally, a multi‐locus approach to identification yields better results due to increased discriminatory power (CBOL Plant Working Group, 2009; Burgess et al., 2011 and as reviewed in Bell et al., 2016).

Mixed origin, environmental samples, such as pollen, are characterized by the presence of DNA from different organisms that may or may not be degraded. Next‐generation sequencing (NGS) technologies now allow high‐throughput sequencing of complex DNA libraries (Liu et al., 2012) including pollen (Bell et al., 2017; Keller et al., 2015; Kraaijeveld et al., 2015; Richardson, Lin, Quijia, et al., 2015; Sickel et al., 2015). Coupling together NGS and DNA barcoding (metabarcoding) could improve on the identification assessments of pollen compared to traditional microscopic identification methods employed by palynology.

In this study, we investigated the possibility of using a historical bee collection as a pollen source for ITS1, ITS2 and *rbcL* metabarcoding and examined the usefulness of this approach to identify plant species from small amounts of pollen carried by bee specimens collected over 100 years ago.

## 2 MATERIALS AND METHODS

### 2.1 Pollen sample collection from bee specimens

Selecting an appropriate bee species for this study was not only dependant on the availability of the species within the collection, but also when specimens were collected. Pollen loads of the specimens can also vary depending on whether the individual bee was captured on their way to or on their way from a floral visit. Based on these criteria, *Megachile venusta* Smith (Megachilidae) specimens were selected from the South African National Collection of Insects housed at Biosystematics, Plant Protection Research: Plant Health, of the Agricultural Research Council (ARC), Pretoria, South Africa. The collection is utilized for taxonomic classification of indigenous bee species, and houses type specimens.

*Megachile venusta* is indigenous to southern Africa, and bee specimens used for pollen sampling were collected from different biomes across South Africa over a period of 93 years (1914–2007). Three bee specimens with visible pollen from each decade, starting from the 1910s up to the 2000s, were selected for inclusion in this study. No specimens with visible pollen were available for the 1930s or 1950s, and these decades are thus not represented here. Only one specimen was available and included for the 1940s. Accession information of the bee specimens used in this study is provided in Supporting Information Table S1.

Pollen samples from the selected *M. venusta* specimens were removed from bee abdomens using sterile micropipette tips dipped in sterilized glycerol while viewing specimens with a stereo dissection microscope (SteREO Discovery.V8 microscope, Carl Zeiss Microscopy GmbH, Jena, Germany). Care had to be taken when working with the old, fragile bee specimens. Each pollen sample was transferred to a sterile 1.5‐ml Eppendorf tube and pollen crushed with the micropipette tip while still under magnification. The micropipette tip for each sample was left inside the tube after scraping off the pollen to include any pollen that inadvertently entered the micropipette tip during scraping.

### 2.2 DNA extraction

To ensure that grains were ruptured evenly across taxa, the DNA extraction protocol was optimized prior to the extraction of pollen samples from historic bee specimens. Both fresh pollen, arbitrarily collected from plants growing at the ARC Biotechnology Platform's grounds at Onderstepoort, Pretoria, and historical pollen on bee specimens were used as test samples for pollen extraction optimization. The following commercial kits were tested: QIAamp DNA Micro Kit (Qiagen, Hilden, Germany), DNeasy® Plant Mini Kit (Qiagen) and Nucleospin® DNA Trace Kit (Macherey‐Nagel GmbH & Co. KG, Düren, Germany). All kits were used according to manufacturer's protocols. In the first experiment, three fresh pollen mixtures were used. Each of these three test samples was divided into two and extracted in parallel using the kits mentioned above, with one reaction subjected to 3 mm steel bead disruption using a TissueLyser II (Qiagen). The other reaction was not subjected to disruption.

In the second optimization experiment, pollen samples collected from six different bees were selected for DNA extraction optimization based on sample age. Bee specimen pollen loads did not vary significantly in size when judged by eye, but actual pollen grain count was not taken into account in selection. Two bee specimens were selected from three different decades, respectively (1980s, 1960s and 2000s). Pollen samples from each decade were used for DNA extraction with the DNeasy® Plant Mini Kit (Qiagen), one sample with and one sample without bead disruption. Bead disruption was performed for 2 min at 25 Hz, with addition of lysis buffers both before and after disruption in different samples. Direct amplification from the pollen template was also tested on fresh pollen, as previously performed by Petersen, Johansen, and Seberg (1996).

DNA from pollen collected from 22 *M. venusta* (Supporting Information Table S1) was extracted using the DNeasy^®^ Plant Mini Kit (Qiagen) without bead disruption, as this method produced the most consistent results. Lysis buffer AP1 and Proteinase K (0.2 mg/ml) were added directly to the Eppendorf tubes containing the micropipette tips used for scraping pollen off the bees. Before transferral of the lysate to the QIAshredder Mini Spin Columns, the micropipette tips were carefully removed using a pair of sterile forceps and excess liquid expelled with a micropipette. In all cases, equipment was cleaned with 10% bleach and 70% ethanol solutions between sampling to avoid cross‐contamination. The remainder of the DNA extraction was performed following the manufacturer's protocol, with the elution step using the protocol recommendation for increasing DNA yield with a minor modification: The 20 μl eluate was reapplied to the DNeasy Mini Spin Column and eluted for a second time.

### 2.3 Barcode amplification and sequencing

Three regions were targeted for DNA barcoding to identify pollen origins, namely, ITS1, ITS2 and *rbcL*, with primers available in the literature for ITS1 and ITS2 (White, Bruns, Lee, & Taylor, 1990) and *rbcL* (de Vere et al., 2012; Fazekas et al., 2008; Kress, Wurdack, Zimmer, Weigt, & Janzen, 2005). Primers were modified by adding overhang adapters compatible with the standard Illumina indexing PCR as described in the Illumina *16S Metagenomic Sequencing Library Preparation Guide* (Illumina, 2013). The modified primer sequences are given in Table 1. Amplification of the *rbcL* region was poor, despite extensive optimization. Two reverse primers were tested for *rbcL* due to poor amplification and sequencing results. Amplification products using primer rbcLajf634R_Tag_IL produced some sequence reads, whereas sequencing products after amplification with rbcLr506_Tag_IL did not produce usable results.

**Table 1:** Primer sequences for ITS1 and ITS2 barcodes with the added Illumina adapter overhangs. Primer sequences were obtained from White et al. (1990), Kress et al. (2005), Fazekas et al. (2008), and de Vere et al. (2012)**.** Illumina adapter target sequences (indicated in bold and underlined) were used in accordance with the workflow from the Illumina 16S Metagenomics protocol (Illumina, 2013). These adapter targets allow Nextera indexing and Illumina adapter addition through PCR

Barcode region	Primer name	Primer sequence (5’ to 3’)
ITS1	ITS5F_Tag_IL	**TCG TCG GCA GCG TCA GAT GTG TAT AAG AGA CAG **GGA AGT AAA AGT CGT AAC AAG
	ITS2R_Tag_IL	**GTC TCG TGG GCT CGG AGA TGT GTA TAA GAG ACA G**GC TGC GTT CTT CAT CGA TGC
ITS2	ITS3F_Tag_IL	**TCG TCG GCA GCG TCA GAT GTG TAT AAG AGA CAG **GCA TCG ATG AAG AAC GCA GC
	ITS4R_Tag_IL	**GTC TCG TGG GCT CGG AGA TGT GTA TAA GAG ACA G**TC CTC CGC TTA TTG ATA TGC
*rbcL*	rbcLF_Tag_IL	**TCG TCG GCA GCG TCA GAT GTG TAT AAG AGA CAG **ATG TCA CCA CAA ACA GAG ACT
	rbcLajf634R_Tag_IL	**GTC TCG TGG GCT CGG AGA TGT GTA TAA GAG ACA G**GA AAC GGT CTC TCC AAC GCA T
	rbcLr506_Tag_IL	**GTC TCG TGG GCT CGG AGA TGT GTA TAA GAG ACA G**AG GGG ACG ACC ATA CTT GTT CA

After optimization, barcode amplification reactions for both ITS1 and ITS2 consisted of a final concentration of 0.5 μM of each primer, 200 μM dNTPs, 1X Phusion^®^ High‐Fidelity Buffer, 0.02 U/μl Phusion^®^ High‐Fidelity DNA Polymerase (Thermo Scientific, Waltham, MA, USA) and 5 μl DNA template, irrespective of DNA concentration. Reaction volumes were adjusted to a final reaction volume of 50 μl with Milli‐Q^®^ H_2_O (Merck Millipore, KGaA, Darmstadt, Germany). PCR conditions were as follows: 98°C for 3 min, followed by 30 cycles of 98°C for 7 s, 65°C for 30 s and 72°C for 30 s, with a final step of 72°C for 10 min. Negative controls were included in PCR‐based steps.

Amplified barcodes were visualized using 2% agarose gel electrophoresis. ITS1 and ITS2 barcode sizes differ between plant taxa and ranged between 100 and 700 bp (Yao et al., 2010). The *rbcL* barcode ranged between 500 and 700 bp (Burgess et al., 2011; Fazekas et al., 2008). Amplicons were purified with the QIAamp^®^ MinElute™ PCR Purification Kit (Qiagen). Reapplication of the eluate was performed to increase DNA concentration. DNA quantification was done using a Qubit^®^ 2.0 Fluorometer (Invitrogen, Life Technologies, Carlsbad, CA, USA) and the Qubit^®^ dsDNA High Sensitivity Assay Kit (Invitrogen, Life Technologies).

Sequencing libraries were prepared according to the Nextera XT (Illumina, Inc. San Diego, CA, USA) preparation protocol. Nextera XT indexes were used to multiplex the individual samples and barcodes. Sequencing of a single multiplexed sample was performed using the MiSeq Reagent Kit v3 (2 X 300 bp paired‐end, Illumina, Inc.) on a MiSeq sequencer (Illumina, Inc.) at the ARC Biotechnology Platform, South Africa.

### 2.4 Bioinformatics and sample analyses

Sample demultiplexing was done using MiSeq Reporter v2.5.1 by separating the samples on perfect index matches. Quality and adapter trimming of reads were done using Trimmomatic 0.33 (Bolger, Lohse, & Usadel, 2014) using the script provided in Supporting Information Appendix S2. All sequences with a length below 50 were discarded, and a quality score of 20 was applied for bases with a sliding window of four bases. Nextera adapters were trimmed from sequences in this same step. Reads that passed quality trimming were merged in MacQiime 1.9.1–20150604 (Caporaso et al., 2010). Generalized linear models were used to test the relationship between the number of reads obtained post quality trimming and the age of the sample and quality score (Q‐scores, prediction of the probability of an error in base calling) of sequences produced. The average Phred value (Q‐score) for each sample was calculated from the raw ITS1 and ITS2 fragments before adaptor trimming. For each sample, a Phred score was calculated for each base position across all the fragments obtained for a sample. The average of these across the fragment was then calculated. Q‐scores were calculated for forward and reverse sequences separately and then averaged to provide a single Q‐score for each sample. The number of ITS1 and ITS2 reads was fitted as response and the age of the sample and Q‐score as explanatory variables. A negative binomial regression analysis was performed, as the data were not normally distributed and there was evidence of overdispersion. Model fit was assessed using a likelihood ratio test. Analyses were performed with R 3.5.0 (the R project for statistical computing) with a significance level of *p* < 0.05.

No curated, plant sequence database for ITS1 was available at the time of data analyses. A reference database was therefore constructed by downloading all Viridiplantae sequences for ITS from GenBank using custom Python scripts (Supporting Information Appendix S1). A Hidden Markov modeller, ITSx 1.0.11 (Bengtsson‐Palme et al., 2013), was used to detect ITS1 and ITS2 in the complete ITS sequences downloaded from GenBank and to exclude any sequences detected as anything other than bryophytes, chlorophytes, marchantiophytes and tracheophytes. Pollen samples were taxonomically classified against this downloaded reference sequence database using the “rdp” option (*c* = 0.80) in *assign_taxonomy.py* in MacQiime 1.9.1–20150604 (Caporaso et al., 2010), which used RDP Classifier 2.2 (Wang, Garrity, Tiedje, & Cole, 2007). ITS2 sequence data were analysed with the annotated and curated ITS2 database created by Sickel et al. (2015) using their published bioinformatics pipeline. Taxa represented in a proportion fewer than 0.1% of the total number of reads per sample were discarded (Sickel et al., 2015).

Only reads identified as plant taxa were used to determine species richness. To determine whether samples were sequenced to a sufficient depth to identify all possible plant taxa in the sample, rarefaction curves were drawn using the vegan v. 2.3.4 package in R. Taxonomic assignments for each sample were checked for their presence in the area in which bees were originally sampled using the local Plants of Southern Africa (POSA) v. 3.0 database (www.posa.sanbi.org).

## 3 RESULTS

### 3.1 Pollen DNA extraction from historic bee specimens

DNA was extracted from 22 pollen samples from a historic bee collection, though suitable specimens with sufficient pollen load limited sampling. Pollen load varied between specimens and collection dates. The method of specimen collection (netting vs. malaise trap) and subsequent handling may have inadvertently contributed to pollen losses. In *M. venusta*, approximately 25% of the total specimens within the collection had sufficient pollen for sampling, of which a subset was used here.

All pollen DNA concentrations post‐extraction were too low to be within the accurate quantifiable range of the Qubit^®^ assays. This was not unexpected for the small volumes of pollen obtained from bee specimens. PCR results were consequently used as the measure of success of extractions.

### 3.2 Pollen DNA high‐throughput sequencing

A total number of 660,837 high‐quality merged reads were obtained for ITS1 and 1,130,803 for ITS2 after quality, adapter and length trimming across the 22 pollen samples. This is on average 30,038 reads for ITS1 and 51,400 reads for ITS2 per sample, respectively. Only 40,646 reads were obtained for *rbcL* after 2 sequencing runs, with a mean of <2,000 reads per sample. Mean read lengths for both forward and reverse reads for *rbcL* were very close to the range of our length quality cut‐off (forward mean length = 142 bp; reverse mean length = 103 bp). One sample failed to produce any reads, and <200 reads were obtained for four samples. The number of reads obtained per sample for *rbcL* was significantly lower than for ITS1 (*t* = 4.48, *p* < 0.001) and ITS2 (*t* = 4.03, *p* < 0.001). Table 2 provides summary statistics for ITS1, ITS2 and *rbcL* processed reads.

**Table 2:** A summary of merged processed reads for ITS1, ITS2 and *rbcL* after next‐generation sequencing. Numbers indicated are after reads were processed for quality with Q20 filtering, Nextera adapter trimming, fragments discarded that were less than 100 bp in length and forward and reverse reads merged

	ITS1	ITS2	*rbcL*
Sum of total combined reads	660,837	1,130,803	40,646
Mean of total combined reads	30,038	51,400	1,936
Median of combined reads	20,135	24,668	1,570
Standard deviation	27,246	56,826	1,427

The percentage of reads of both ITS1 and ITS2 assigned to the kingdom Viridiplantae varied between samples. Samples consisting of fewer than 1,000 reads that were classified to species level were regarded as unsuccessful and were discarded prior to further analyses. This cut‐off was selected so that rare taxa identified will have at least two reads. The presence of unidentified reads did not influence the identification of plant origins of samples, even though a higher amount of total reads were necessary to reach sequence saturation for plant identification. Identification of *rbcL* reads to plant origins produced very variable results. In 45% of the samples, less than 1,000 reads were produced. Due to the extremely variable nature of amplification and sequencing results, *rbcL* data were not analysed further.

**Figure 1:**
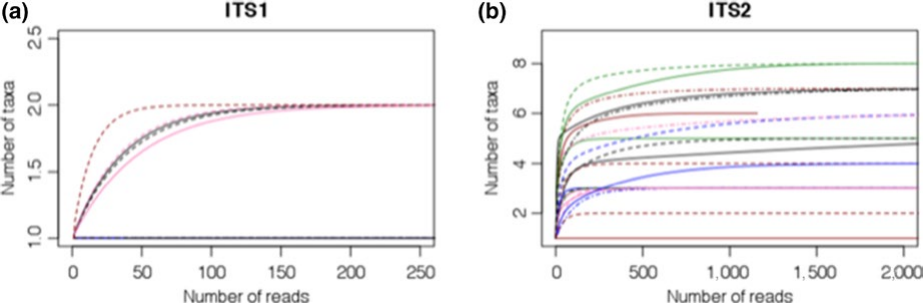
Rarefaction curves for (a) ITS1 and (b) ITS2 samples. ITS1 samples reached sequence saturation at approximately 250 reads, whereas ITS2 samples needed approximately 1,000 to 2,000 high‐quality sequence reads to obtain maximum plant taxon richness per sample. Rarefaction curves were created after taxa representing less than 0.1% of reads per sample were removed. Rarefaction curves without <0.1% reads removed are in available in the Supporting Information

After removal of taxa representing <0.1% of reads per sample, only one or two plant genera per sample for ITS1 could be identified against the sequence reference database generated in this study. Between one and eight plant species were identified per sample using ITS2. Rarefaction curves show that the sequencing depth for all samples was sufficient to obtain maximum taxon richness (Figure 1). When all raw read data are included in rarefaction analyses, a maximum of ten species per sample for ITS2 was reached, with curves still reaching a plateau (Figure S1), thus further indicated that sufficient sequencing depth was reached.

A total of 81.8% of ITS1 samples had more than 1,000 reads identified to Viridiplantae. One of the ITS2 samples only had 1,154 high‐quality, merged reads that could be identified to Viridiplantae, but was still sequenced to saturation, as indicated by the rarefaction curve (Figure 1b). A single ITS2 sample had less than 1,000 reads identified to Viridiplantae and was removed from further analyses, with 95.5% of samples remaining for further analyses.

### 3.3 Plant origins of pollen collected from Megachile venusta specimens

When classifying sequence reads to the ITS1 database, two plant genera (*Helianthus* and *Oryza*) were identified. *Helianthus* was identified in 72.2% of the samples, and both genera were identified in the remaining 27.8% of samples. On average 3.3% (*SD* = 0.25) of reads per sample could only be assigned to the phylum level (Streptophyta) and 50.3% (*SD* = 0.09) of reads remained unidentified at the assignment level of kingdom. Classification to species level was limited with the ITS1 database.

**Figure 2:**
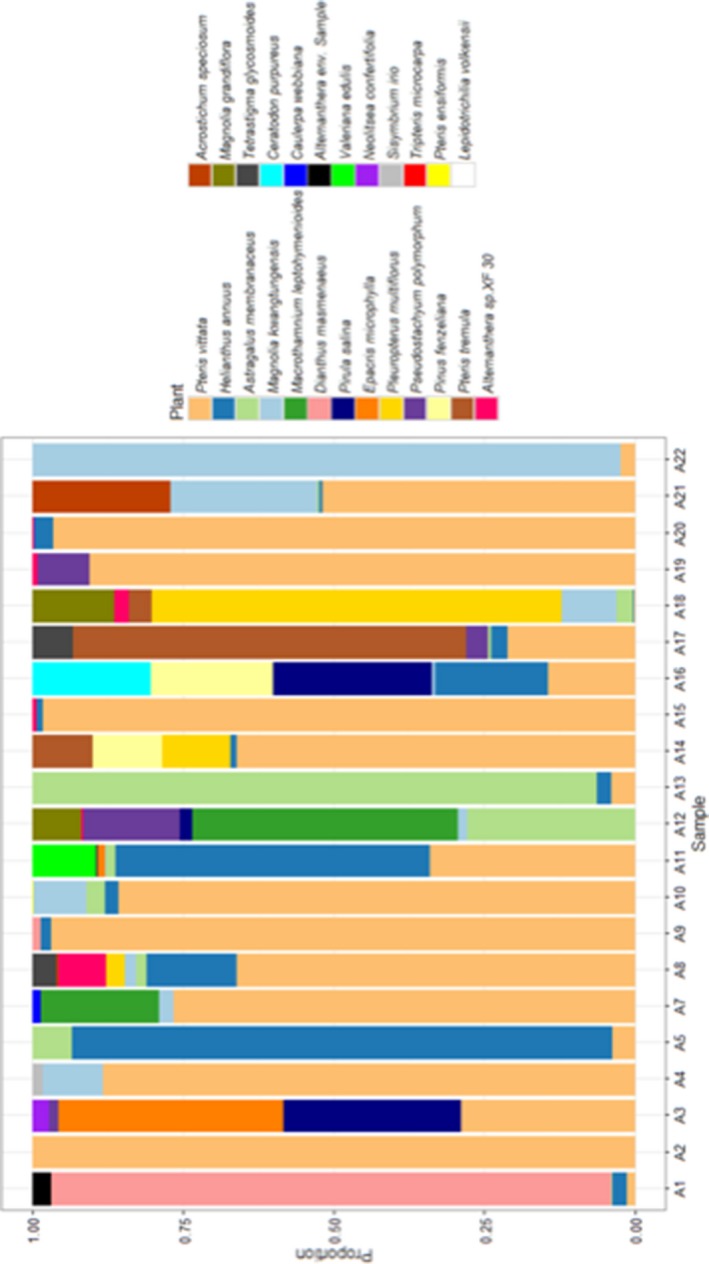
Bar graph representing 22 samples classified with the ITS2 database. The different colours indicate different plant species identified with the sequence database, and in which percentage it was detected in each sample

Classification with the ITS2 database produced classification only up to kingdom in 0.6% of the reads per sample, on average (*SD* = 0.02) and only up to phylum for an average of 68.4% (*SD* = 0.22) of reads per sample. Significantly more lower ranking taxon classifications could be made using the ITS2 database. With the confidence set at the recommended level of 85%, an average of four species, four genera and four families were identified per sample when classifying reads with the ITS2 database. In total, 25 species from 21 different genera could be confidently identified with the ITS2 database. These species belonged to 19 different families, 16 orders and six classes. The five most dominant plant species identified were *Pteris vittata* (34.6%), *Helianthus annuus* (32.4%), *Astragalus membranaceus* (17.2%), *Magnolia kwangtungensis* (3.3%) and *Macrothamnium leptohymenioides* (3.2%). Two algae species, *Caulerpa webbiana* (identified in one sample) and *Pirula salina* (identified in three samples), were identified. A summary of all plant species identified and associated abundance can be seen in Figure 2. However, several sequence reads could not be classified confidently to species level with the ITS2 database. Eight taxa could only be classified to genus level, five to family, two to class and another one to order. Of the eight genera identified, three correspond to prior species‐level classifications, with five genera newly identified (Table 3).

**Table 3:** Viridiplantae taxa that were not classified to species‐level, but to genus and family‐level. Two classes were also identified, Liliopsida and Magnoliopsida, as well as the order Cucurbitales

Family	Genus
Asteraceae^a^	*Helianthus* b
	*Lactuca*
Amaranthaceae^a^	*Amaranthus*
	*Alternanthera* b
Magnoliaceae	*Magnolia* b
Proteaceae	*Macadamia*
Moraceae	*Morus*
Trebouxiaphyceae	*Trebouxia*
Cucurbitaceae^c^	*–*
Fabaceae^a^	*–*
Poaceae^a^	–

a Four of these families have been identified during species‐level classification.

b Taxa also identified during species‐level classification.

c Five taxa identified for which sequence reads could be classified up to family level.

From the different taxa distinguished in the pollen from *M. venusta* samples, data for 15 genera and for only six species were available in the POSA v. 3.0 database. The available plant distribution data overlap well with the geographic origins of the bee samples. For 86.4% of samples, all identified genera occurred within the area where *M. venusta* was sampled and from which pollen was sequenced. Four species in three samples did not have occurrence data in the POSA v 3.0 database. These were all from the Northern Cape Province of South Africa, which is botanically under‐surveyed.

**Figure 3:**
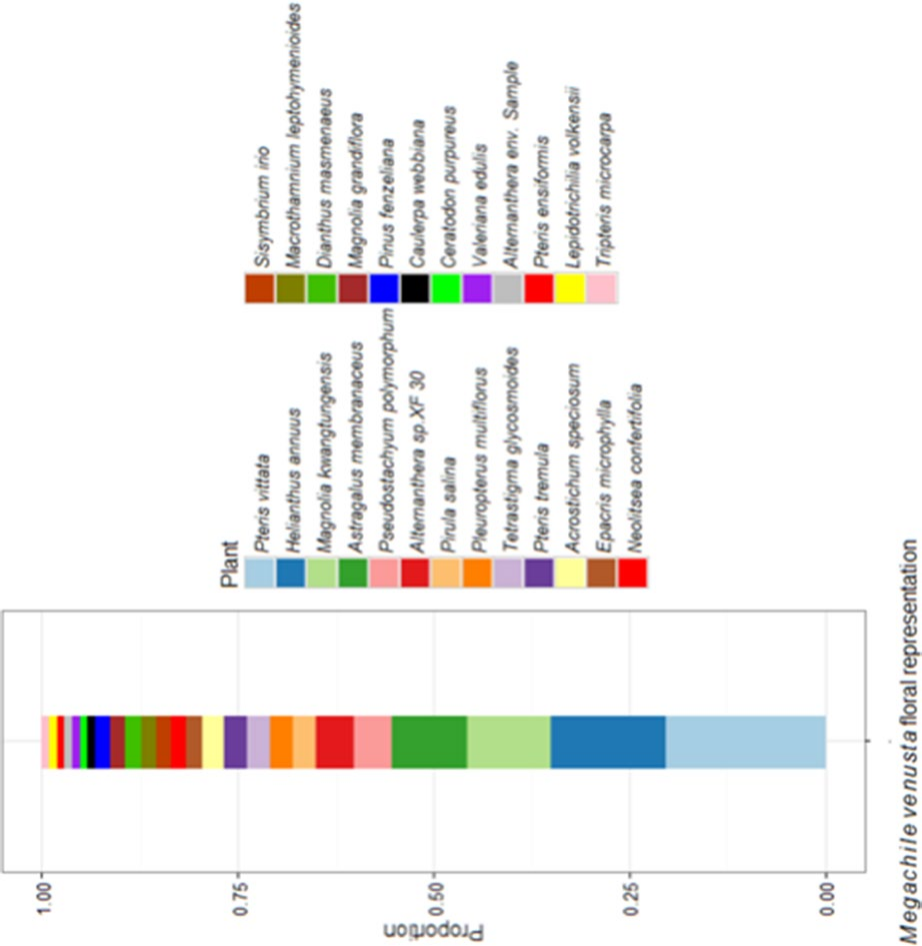
Floral representation of pollen sampled from all *M. venusta* bee specimens. *P. vittata, H. annuus, M. kwangtungensis* and *A. membranaceus* are the most highly represented plant species from pollen of *M. venusta* specimens

Combined ITS2 classification results of all samples from *M. venusta* specimens provide insight into the floral choice of this South African indigenous bee species. The most commonly encountered plant species was *Pteris vittata,* followed by *Helianthus annuus*,* Magnolia kwangtungensis* and *Astragalus membranaceus* (Figure 3).

### 3.4 Effect of sample age on sequencing success

Pollen from as far back as 1914 was successfully metabarcoded in this study. A negative binomial regression was run to determine the relationship between the number of reads obtained post quality trimming in 22 pollen samples and the age of the specimen from which the pollen originated and the Q‐scores obtained for both ITS1 and ITS2 sequences. The age of sample term in generalized linear model, although negative, was non‐significant for both ITS1 (Z = −0.70, *df* = 19, *p* = 0.49) and ITS2 (Z = −1.43, *df* = 19, *p* = 0.15). This suggests that the age of sample did not have a negative impact on pollen barcode amplification. In contrast, *Q*‐scores were a statistically significant predictor of the number of ITS1 (*Z* = 5.66, *df* = 19, *p* < 0.001) and ITS2 (*Z* = 5.47, *df* = 19, *p* < 0.001) reads. This is not surprising given that lower Q‐scores can result in a significant portion of the unreliable reads that would have been removed during quality trimming.

## 4 DISCUSSION

Historic specimen collections can essentially be seen as large, untapped resources of data, especially for plant–pollinator interaction investigations. This is particularly important for species where field observation is difficult. Honeybee pollination is well studied, and the NGS workflow tested in this study could significantly improve knowledge on less well‐studied species. As pollinator specimens in historic collections usually have accompanying metadata, these specimens are invaluable to researchers interested in pollination and change in ecosystems or plant communities over time.

DNA barcoding has been used for years to successfully identify unknown plants (Burgess et al., 2011), and the recent uptake of pollen sequencing by the metabarcoding community (Galimberti et al., 2014; Hawkins et al., 2015; Keller et al., 2015; Kraaijeveld et al., 2015; Richardson, Lin, Sponsler, et al., 2015) has sparked new interest in the topic. In this study, DNA metabarcoding was used to determine the plant origins of limited pollen sampled directly from *M. venusta* bees taken from a historic collection. The pollen exine is exceptionally resilient ensuring DNA contained within the pollen grain maintains its integrity for a very long time, making ancient pollen studies possible (Parducci, Suyama, Lascoux, & Bennett, 2005).

The choice of DNA barcode to identify plant species has been controversial. This has led to the selection of a suite of markers identified as potential candidates (Hollingsworth et al. 2009; Hollingsworth 2011; Hollingsworth, Graham, & Little, 2011; Li, Gao, Poudel, Li, & Forrest, 2011). The utility of these on low concentration, degraded DNA from historical samples, has not been formally assessed. In this study, we examine the utility of three DNA barcode markers (ITS1, ITS2 and *rbcL*) to identify plants visited by pollinators from small amounts of pollen collected from an important bee collection. Sequencing results indicate that historic pollen identification using DNA barcoding on an NGS platform was most successful using ITS1 and ITS2, regardless of limited starting material or the age of the specimen. In contrast, *rbcL* produced variable amplification results between samples. Two different reverse primers were tested for this gene and only amplicons produced using the rbcLajf634F_Tag_IL primer produced sequence results. However, low clustering on the MiSeq flow cells occurred during both sequence runs, with variability between samples. *RbcL* has been successfully used before in pollen barcoding with both Sanger sequencing (Bruni, Galimberti, & Caridi, 2015; Galimberti et al., 2014) and with NGS (Richardson, Lin, Quijia, et al., 2015). However, the amount of pollen used for DNA extraction in these cases was notably higher than that used in this study: 50 mg (Richardson, Lin, Quijia, et al., 2015) and 100 mg (Galimberti et al., 2014). Plastids are maternally inherited in many floral taxa, and they will consequently not be present in all pollen grains (Bennett & Parducci, 2006; Corriveau, Goff, & Coleman, 1990). This poses a false‐negative versus true‐negative PCR amplification problem for a barcoding approach using plastid genes, with uncertainty about what causes the amplification failure. Due to variable amplification and poor sequencing results obtained with the *rbcL* amplicons, this gene does not appear to be a good choice for an affordable, reliable metabarcode workflow for pollen sampled from bee archival specimens in natural history collections.

For plant origin tracing of pollen, rarefaction curves indicate that enough reads were sequenced for ITS1 and ITS2 samples to reach sequence saturation. Only 250 reads per sample were necessary to reach a plateau during rarefaction for ITS1 since only two species were identified using this barcode. For ITS2, between 750 and 2,000 reads were necessary to reach sequence saturation, with the upper limit consistent with previous pollen metabarcoding results (Sickel et al., 2015).

The lower number of plant taxa identified per sample in this study is concordant with the observed floral constancy behaviour in foraging bees (Michener, 2000), where bees tend to visit flowers from plants of the same taxa during one foraging trip as long as this resource remains available. Pollen in this study was sampled directly from bee specimens that were actively foraging during their capture, and therefore, the lower number of plant taxa obtained is not surprising. Sampling pollen from pollen traps (Keller et al., 2015; Richardson, Lin, Quijia, et al., 2015) or honey (Bruni et al., 2015; Hawkins et al., 2015) is expected to yield considerably more plant taxa as these pollen samples originate from multiple bees and cover many foraging trips. Indeed, the plant families identified here using metabarcoding correspond with the foraging information known for *M. venusta*, with the Asteraceae, Fabaceae and Poaceae associations observed for the species in South African (Eardley, 2013).

All plant families identified from the pollen were present in the localities where bee samples were collected. The metabarcoding data indicated the presence of four plant genera in the Northern Cape Province of South Africa which are not currently listed in the POSA database. One of these species, *Helianthus annuus*, is grown in fields in the Northern Cape but was absent from the plant database, probably because this crop is not native to the region. Although we cannot discount the presence of false positives in our data (Bell et al., 2017), the POSA database was last updated in 2012, making its use limited. Only six species identified with the ITS2 database were represented in POSA. This is due to poor species representation in the plant database, or sequence misclassification due to limited representation in the sequence reference database, allowing closely related sequences to be assigned with high enough confidence even though it does not represent the true plant origin. Some of the species identified are not native to South Africa, such as *Magnolia* spp., and *Pseudostachyum polymorphum.* A simple Internet search for *Magnolia* in South Africa, however, revealed that *Magnolia grandiflora* is readily traded as an ornamental plant.

Species of interest were *Pteris vittata* and *Pteris ensiformis,* a genus of fern that was present in all ITS2 samples. Ferns do not produce pollen and are not known to have any animal involvement in reproduction. Ferns usually produce large numbers of spores that are easily dispersed into the environment. These identifications could be due to environmental spore contamination. However, alternative explanations for the detection of ferns are that sequences could also have been misclassified or the sequences representing this genus in the underlying database could potentially have been incorrectly assigned. However, upon investigation of the underlying entries in GenBank, it was found that five entries from the same batch are identical to fungal sequences, which could indicate that these samples were misrepresented in NCBI. When those sequences were removed from the database, no more classification of *Pteris* occurred. This demonstrates the importance of the quality of the reference sequence database. The same entries are also present in the ITS2 database but were not removed prior to classification, as a retraining of the classifier would have needed to be done.

The identification from a reference database will also only occur if the specific species was barcoded before, correctly classified and phylogenetically assigned. The International Barcode of Life (iBOL, www.ibol.org) project aims to achieve this. In this study of pollinator–plant interactions in a hyper‐diverse region, more confidence should be placed in higher‐level classifications, with family‐level interpretation likely being the most accurate.

## 5 CONCLUSION

Pollen metabarcoding of historic collections opens up the possibility to reconstruct the plant communities that pollinators visited in the past. By doing this, changes in their floral choice can be tracked both temporally and spatially, giving insight in how different environmental factors affect them. Understanding the influences of factors such as climate change and land use change on plant–pollinator interactions could prove vital in the conservation of vulnerable species, both plant and animal. Small amounts of pollen sampled from historic *M. venusta* bees, dating back 103 years, were successfully used for DNA extraction, amplification and sequencing on an NGS platform. This shows that museum collections could indeed be a valuable resource in pollinator–plant studies. DNA metabarcoding was used to identify the plant origins in pollen. Using ITS2 as a barcode provided much better resolution for plant classification than ITS1. Multi‐locus approaches to DNA barcoding for plants are recommended, and ITS1 data should therefore be considered with ITS2. Species‐level plant classification is possible with ITS2, but without a comprehensive local plant sequence reference database, family‐based interpretations are more reliable.

## AUTHOR CONTRIBUTIONS

A. Gous, C.D. Eardley, Z.H. Swanevelder and S. Willows‐Munro conceived and designed the study. A. Gous performed the acquisition, analyses and interpretation of data. A. Gous and S. Willows‐Munro drafted the manuscript, and Z.H. Swanevelder and C.D. Eardley critically revised the manuscript for intellectual content. All authors approved the final version of the manuscript to be published.

## DATA ACCESSIBILITY

DNA sequences: All metabarcoding data are available to the European Nucleotide Archive (ENA) under project accession PRJEB14178 (https://www.ebi.ac.uk/ena/data/view/PRJEB14178).

## References

[eva12707-bib-0001] Bell, K. L. , de Vere, N. , Keller, A. , Richardson, R. T. , Gous, A. , Burgess, K. S. , & Brosi, B. J. (2016). Pollen DNA barcoding: Current applications and future prospects. Genome, 59, 629–640. 10.1139/gen-2015-0200 27322652

[eva12707-bib-0002] Bell, K. L. , Fowler, J. , Burgess, K. S. , Dobbs, E. K. , Gruenewald, D. , Lawley, B. , … Brosi, B. J. (2017). Applying pollen DNA metabarcoding to the study of plant–pollinator interactions. Applications in Plant Sciences, 5, 1600124 10.3732/apps.1600124 PMC5499302 28690929

[eva12707-bib-0003] Bengtsson‐Palme, J. , Ryberg, M. , Hartmann, M. , Branco, S. , Wang, Z. , Godhe, A. , … Nilsson, R. H. (2013). Improved software detection and extraction of ITS1 and ITS2 from ribosomal ITS sequences of fungi and other eukaryotes for analysis of environmental sequencing data. Methods in Ecology and Evolution, 4, 914–919. 10.1111/2041-210X.12073

[eva12707-bib-0004] Bennett, K. D. , & Parducci, L. (2006). DNA from pollen: Principles and potential. The Holocene, 16, 1031–1034. 10.1177/0959683606069383

[eva12707-bib-0006] Bolger, A. M. , Lohse, M. , & Usadel, B. (2014). Trimmomatic: A flexible trimmer for Illumina sequence data. Bioinformatics, 30, 2114‐2120. 24695404 10.1093/bioinformatics/btu170 PMC4103590

[eva12707-bib-0008] Bruni, I. , Galimberti, A. , Caridi, L. , et al. (2015). A DNA barcoding approach to identify plant species in multiflower honey. Food Chemistry, 170, 308–315. 10.1016/j.foodchem.2014.08.060 25306350

[eva12707-bib-0009] Burgess, K. S. , Fazekas, A. J. , Kesanakurti, P. R. , Graham, S. W. , Husband, B. C. , Newmaster, S. G. , … Barrett, S. C. H. (2011). Discriminating plant species in a local temperate flora using the *rbcL*+*matK* DNA barcode: Barcoding plants in a local flora. Methods in Ecology and Evolution, 2, 333–340. 10.1111/j.2041-210X.2011.00092.x

[eva12707-bib-0010] Caporaso, J. G. , Kuczynski, J. , Stombaugh, J. , Bittinger, K. , Bushman, F. D. , Costello, E. K. , … Knight, R. (2010). QIIME allows analysis of high‐throughput community sequencing data. Nature Methods, 7, 335–336. 10.1038/nmeth.f.303 20383131 PMC3156573

[eva12707-bib-0011] CBOL Plant Working Group (2009). A DNA barcode for land plants. Proceedings of the National Academy of Sciences of the United States of America, 106, 12794–12797. 10.1073/pnas.0905845106 19666622 PMC2722355

[eva12707-bib-0012] Chen, S. , Yao, H. , Han, J. , Liu, C. , Song, J. , Shi, L. , … Leon, C. (2010). Validation of the ITS2 region as a novel DNA barcode for identifying medicinal plant species. PLoS One, 5, e8613. 20062805 10.1371/journal.pone.0008613 PMC2799520

[eva12707-bib-0013] Corriveau, J. L. , Goff, L. J. , & Coleman, A. W. (1990). Plastid DNA is not detectable in the male gametes and pollen tubes of an angiosperm (*Antirrhinum majus*) that is maternal for plastid inheritance. Current Genetics, 17, 439–444.

[eva12707-bib-0014] Dafni, A. (1992). Pollination ecology: A practical approach. Oxford, UK: Oxford University Press.

[eva12707-bib-0015] Daily, G. C. , Alexander, S. , Ehrlich, P. R. , Goulder, L. , Lubchenco, J. , Matson, P. A. , … Woodwell, G. M. (1997). Ecosystem services: Benefits supplied to human societies by natural ecosystems. Issues in Ecology, 2, 1–18.

[eva12707-bib-0016] DeVere, N. , Rich, T. C. G. , Ford, C. R. , Trinder, S. A. , Long, C. , Moore, C. W. , … Wilkinson, M. J. (2012). DNA barcoding the native flowering plants and conifers of Wales. PLoS One, 7, e37945. 22701588 10.1371/journal.pone.0037945 PMC3368937

[eva12707-bib-0017] Dong, W. , Xu, C. , Li, C. , Sun, J. , Zuo, Y. , Shi, S. , … Zhou, S. (2015). *ycf1*, the most promising plastid DNA barcode of land plants. Scientific Reports, 5, 8348 10.1038/srep08348 25672218 PMC4325322

[eva12707-bib-0018] Eardley, C. (2013). A taxonomic revision of the southern African leaf‐cutter bees, *Megachile* Latreille *sensu stricto* and *Heriadopsis* Cockerell (Hymenoptera: Apoidea: Megachilidae). Zootaxa, 3601, 1–134. 24614086 10.11646/zootaxa.3601.1.1

[eva12707-bib-0020] Fazekas, A. J. , Burgess, K. S. , Kesanakurti, P. R. , Graham, S. W. , Newmaster, S. G. , Husband, B. C. , … Barrett, S. C. H. (2008). Multiple multilocus DNA barcodes from the plastid genome discriminate plant species equally well. PLoS One, 3, e2802. 18665273 10.1371/journal.pone.0002802 PMC2475660

[eva12707-bib-0021] Ferri, G. , Corradini, B. , Ferrari, F. , Santunione, A. L. , Palazzoli, F. , & Alu’, M. (2015). Forensic botany II, DNA barcode for land plants: Which markers after the international agreement? Forensic Science International: Genetics, 15, 131–136. 10.1016/j.fsigen.2014.10.005 25457632

[eva12707-bib-0022] Galimberti, A. , De Mattia, F. , Bruni, I. , Scaccabarozzi, D. , Sandionigi, A. , Barbuto, M. , … Labra, M. (2014). A DNA barcoding approach to characterize pollen collected by honeybees. PLoS One, 9, e109363. 25296114 10.1371/journal.pone.0109363 PMC4190116

[eva12707-bib-0025] Hargreaves, A. L. , Johnson, S. D. , & Nol, E. (2004). Do floral syndromes predict specialization in plant pollination systems? An experimental test in an “ornithophilous” African Protea. Oecologia, 140, 295–301. 10.1007/s00442-004-1495-5 15168105

[eva12707-bib-0027] Hawkins, J. , de Vere, N. , Griffith, A. , Ford, C. R. , Allainguillaume, J. , Hegarty, M. J. , … Adams‐Groom, B. (2015). Using DNA metabarcoding to identify the floral composition of honey: A new tool for investigating honey bee foraging preferences. PLoS One, 10, e0134735. 26308362 10.1371/journal.pone.0134735 PMC4550469

[eva12707-bib-0028] Hebert, P. D. N. , Cywinska, A. , Ball, S. L. , & deWaard, J. R. (2003). Biological identifications through DNA barcodes. Proceedings of the Royal Society B: Biological Sciences, 270, 313–321. 10.1098/rspb.2002.2218 PMC1691236 12614582

[eva12707-bib-0029] Hollingsworth, P. M. (2011). Refining the DNA barcode for land plants. Proceedings of the National Academy of Sciences USA, 108, 19451–19452. 10.1073/pnas.1116812108 PMC3241790 22109553

[eva12707-bib-0030] Hollingsworth, M. L. , Clark, A. A. , Forrest, L. L. , Richardson, J. , Pennington, R. T. , Long, D. G. , ... Hollingsworth, P. M. (2009). Selecting barcoding loci for plants: Evaluation of seven candidate loci with species‐level sampling in three divergent groups of land plants. Molecular ecology resources, 9, 439–457. 21564673 10.1111/j.1755-0998.2008.02439.x

[eva12707-bib-0031] Hollingsworth, P. M. , Graham, S. W. , & Little, D. P. (2011). Choosing and using a plant DNA barcode. PLoS One, 6, e19254. 21637336 10.1371/journal.pone.0019254 PMC3102656

[eva12707-bib-0032] Illumina (2013). 16S metagenomic sequencing library preparation.

[eva12707-bib-0033] Johnson, S. D. (1997). Pollination ecotypes of *Satyrium hallackii* (Orchidaceae) in South Africa. Botanical Journal of the Linnean Society, 123, 225–235. 10.1111/j.1095-8339.1997.tb01415.x

[eva12707-bib-0034] Keller, A. , Danner, N. , Grimmer, G. , Ankenbrand, M. , von der Ohe, K. , von der Ohe, W. , … Steffan‐Dewenter, I. (2015). Evaluating multiplexed next‐generation sequencing as a method in palynology for mixed pollen samples. Plant Biology, 17, 558–566. 10.1111/plb.12251 25270225

[eva12707-bib-0035] Klein, A.‐M. , Vaissiere, B. E. , Cane, J. H. , Steffan‐Dewenter, I. , Cunningham, S. A. , Kremen, C. , & Tscharntke, T. (2007). Importance of pollinators in changing landscapes for world crops. Proceedings of the Royal Society B: Biological Sciences, 274, 303–313. 10.1098/rspb.2006.3721 PMC1702377 17164193

[eva12707-bib-0037] Kraaijeveld, K. , de Weger, L. A. , Ventayol García, M. , Buermans, H. , Frank, J. , Hiemstra, P. S. , & den Dunnen, J. T. (2015). Efficient and sensitive identification and quantification of airborne pollen using next‐generation DNA sequencing. Molecular Ecology Resources, 15, 8–16. 10.1111/1755-0998.12288 24893805

[eva12707-bib-0038] Kremen, C. , Williams, N. M. , Aizen, M. A. , Gemmill‐Herren, B. , LeBuhn, G. , Minckley, R. , … Ricketts, T. H. (2007). Pollination and other ecosystem services produced by mobile organisms: A conceptual framework for the effects of land‐use change. Ecology Letters, 10, 299–314. 10.1111/j.1461-0248.2007.01018.x 17355569

[eva12707-bib-0039] Kress, W. J. , & Erickson, D. L. (2008). DNA barcodes: Genes, genomics, and bioinformatics. Proceedings of the National Academy of Sciences of the United States of America, 105, 2761–2762. 10.1073/pnas.0800476105 18287050 PMC2268532

[eva12707-bib-0040] Kress, W. J. , García‐Robledo, C. , Uriarte, M. , & Erickson, D. L. (2015). DNA barcodes for ecology, evolution, and conservation. Trends in Ecology & Evolution, 30, 25–35. 10.1016/j.tree.2014.10.008 25468359

[eva12707-bib-0041] Kress, W. J. , Wurdack, K. J. , Zimmer, E. A. , Weigt, L. A. , & Janzen, D. H. (2005). Use of DNA barcodes to identify flowering plants. Proceedings of the National Academy of Sciences of the United States of America, 102, 8369–8374. 10.1073/pnas.0503123102 15928076 PMC1142120

[eva12707-bib-0042] Li, Y. , Gao, L. M. , Poudel, R. C. , Li, D. Z. , & Forrest, A. (2011). High universality of matK primers for barcoding gymnosperms. Journal of systematics and evolution, 49, 169–175.

[eva12707-bib-0043] Liu, L. , Li, Y. , Li, S. , Hu, N. , He, Y. , Pong, R. , … Law, M. (2012). Comparison of next‐generation sequencing systems. Journal of Biomedicine and Biotechnology, 2012, 1–11. 10.1155/2012/251364 22829749 PMC3398667

[eva12707-bib-0044] Michener, C. D. (2000). The bees of the world. Baltimore, Maryland: Johns Hopkins University Press.

[eva12707-bib-0045] Mullins, J. , & Emberlin, J. (1997). Sampling pollens. Journal of Aerosol Science, 28, 365–370. 10.1016/S0021-8502(96)00439-9

[eva12707-bib-0046] Parducci, L. , Suyama, Y. , Lascoux, M. , & Bennett, K. D. (2005). Ancient DNA from pollen: A genetic record of population history in Scots pine. Molecular Ecology, 14, 2873–2882. 10.1111/j.1365-294X.2005.02644.x 16029485

[eva12707-bib-0047] Pennisi, E. (2000). Taxonomic revival. Science, 289, 2306–2308. 10.1126/science.289.5488.2306 11041799

[eva12707-bib-0048] Petersen, G. , Johansen, B. , & Seberg, O. (1996). PCR and sequencing from a single pollen grain. Plant Molecular Biology, 31, 189–191. 10.1007/BF00020620 8704154

[eva12707-bib-0049] Richardson, R. T. , Lin, C.‐H. , Quijia, J. O. , Riusech, N. S. , Goodell, K. , & Johnson, R. M. (2015). Rank‐based characterization of pollen assemblages collected by honey bees using a multi‐locus metabarcoding approach. Applications in Plant Sciences, 3, 1500043. 10.3732/apps.1500043 PMC4651628 26649264

[eva12707-bib-0050] Richardson, R. T. , Lin, C.‐H. , Sponsler, D. B. , Quijia, J. O. , Goodell, K. , & Johnson, R. M. (2015). Application of ITS2 metabarcoding to determine the provenance of pollen collected by honey bees in an agroecosystem. Applications in Plant Sciences, 3, 1400066. 10.3732/apps.1400066 PMC4298230 25606352

[eva12707-bib-0052] Sickel, W. , Ankenbrand, M. J. , Grimmer, G. , Holzschuh, A. , Härtel, S. , Lanzen, J. , … Keller, A. (2015). Increased efficiency in identifying mixed pollen samples by meta‐barcoding with a dual‐indexing approach. BMC, Ecology, 15 10.1186/s12898-015-0051-y PMC4509727 26194794

[eva12707-bib-0053] Valentini, A. , Miquel, C. , & Taberlet, P. (2010). DNA barcoding for honey biodiversity. Diversity, 2, 610–617. 10.3390/d2040610

[eva12707-bib-0054] Wang, Q. , Garrity, G. M. , Tiedje, J. M. , & Cole, J. R. (2007). Naive Bayesian classifier for rapid assignment of rRNA sequences into the new bacterial taxonomy. Applied and Environmental Microbiology, 73, 5261–5267. 10.1128/AEM.00062-07 17586664 PMC1950982

[eva12707-bib-0055] Wang, X.‐C. , Liu, C. , Huang, L. , Bengtsson‐Palme, J. , Chen, H. , Zhang, J.‐H. , … Li, J.‐Q. (2015). ITS1: A DNA barcode better than ITS2 in eukaryotes? Molecular Ecology Resources, 15, 573–586. 10.1111/1755-0998.12325 25187125

[eva12707-bib-0056] Wester, P. , Stanway, R. , & Pauw, A. (2009). Mice pollinate the Pagoda Lily, *Whiteheadia bifolia* (Hyacinthaceae) — First field observations with photographic documentation of rodent pollination in South Africa. South African Journal of Botany, 75, 713–719. 10.1016/j.sajb.2009.07.005

[eva12707-bib-0057] White, T. J. , Bruns, T. , Lee, S. , & Taylor, J. (1990). Amplification and direct sequencing of fungal ribosomal RNA genes for phylogenetics In Innis M. A., Gelfand D. H., Sninsky J. J. & White T. J. (Eds.), PCR protocols: A guide to methods and applications (pp. 315–332). San Diego, CA: Academic Press.

[eva12707-bib-0058] Wilcock, C. , & Neiland, R. (2002). Pollination failure in plants: Why it happens and when it matters. Trends in Plant Science, 7, 270–277. 10.1016/S1360-1385(02)02258-6 12049924

[eva12707-bib-0059] Williams, N. M. , & Kremen, C. (2007). Resource distributions among habitats determine solitary bee offspring production in a mosaic landscape. Ecological Applications, 17, 910–921. 10.1890/06-0269 17494406

[eva12707-bib-0061] Yao, H. , Song, J. , Liu, C. , Luo, K. , Han, J. , Li, Y. , … Chen, S. (2010). Use of ITS2 region as the universal DNA barcode for plants and animals. PLoS ONE, 5, e13102. 20957043 10.1371/journal.pone.0013102 PMC2948509

