# Supplementary material for: Plant–pollinator interactions over time: Pollen metabarcoding from bees in a historic collection
Source: Evol Appl. 2018 Nov 13;12(2):187–97. doi: 10.1111/eva.12707 (PMC6346658; doi:10.1111/eva.12707)
Supplement: Supplementary file 1 [file EVA-12-187-s001.docx]

**Supporting information:**

**Figure S1: Rarefaction curves for a) ITS1 and b) ITS2 samples.** Rarefaction curves were created on all reads, prior to removal of 0.1% rare taxa per sample. No difference was observed for ITS1, but an increase of two species was observed in the maximum number of species per sample for sITS2.
