# Supplementary material for: Plant–pollinator interactions over time: Pollen metabarcoding from bees in a historic collection
Source: Evol Appl. 2018 Nov 13;12(2):187–97. doi: 10.1111/eva.12707 (PMC6346658; doi:10.1111/eva.12707)
Supplement: Supplementary file 2 [file EVA-12-187-s002.docx]

| Table S1. *Megachile venusta* bee specimens used for pollen sample collection in this study. Bees were obtained from the National Insect Collection housed at the ARC’s Biosystematics, Pretoria, South Africa. Collection information, such as the date, province, GPS coordinates and nearest town are given for each sample, where available. The total number of reads obtained post quality trimming is given first for ITS1, and then for ITS2. The most prevalent genus detected with the ITS1 marker, and then the ITS2 marker is also given in the last column. | | | | | | | |
| --- | --- | --- | --- | --- | --- | --- | --- |
| **Bee collection identifier^1^** | **Sample identifier in this study** | **Bee collection date** | **Province^2^** | **GPS** | **Bee collection description** | **Number of reads obtained for ITS1/ITS2** | **Most prevalent genus detected with ITS1/ITS2** |
| HYMA05682 | A1 | 10.12.1916 | Transvaal (Gauteng) | 25.43S 28.11E | Pretoria | 17,623/8,169 | *Helianthus*/ *Dianthus* |
| HYMA05679 | A2 | 30.10.1914 | Orange Free State (Free State) | 29.07S27.28E | Modderpoort | 7,513/12,621 | *Helianthus*/ *Pteris* |
| HYMA05680 | A3 | 05.11.1914 | Orange Free State (Free State) | 29.27S26.13E | Bloemfontein | 19,408/42,679 | *Helianthus*/ *Pseudostachyum* |
| HYMA05677/1 | A4 | 18.10.1921 | North West | 27.32S 24.48E | Taung | 11,953/16,588 | *Helianthus*/ *Pteris* |
| HYMA05678 | A5 | 11.1921 | Transvaal (Mpumalanga) | 25.28S 30.59E | Nelspruit (Mbombela) | 20,862/12,429 | *Helianthus*/ *Helianthus* |
| HYMA05677/2 | A6 | 18.10.1921 | North West | 27.32S 24.48E | Taung | 4,002/6,479 | *Helianthus*/ *Helianthus* |
| HYMA27297/1 | A7 | 12.1948 | Eastern Cape | 33.50S 25.34E | Redhouse near Port Elizabeth | 14,047/61,883 | *Helianthus*/ *Pteris* |
| HYMA05723 | A8 | 21.05.1969 | Transvaal(Mpumalanga) | 25.21S 31.53E | Kruger National Park at Crocodile bridge | 18,495/140,145 | *Helianthus*/ *Alternanthera* |
| HYMA05505 | A9 | 23.09.1962 | Transvaal (North West) | 25.39S 26.41E | Swartruggens | 23,867/33,769 | *Helianthus*/ *Pteris* |
| HYMA05503 | A10 | 5.03.1963 | Natal (KwaZulu-Natal) | 29.00S 29.53E | Estcourt | 13,667/17,052 | *Helianthus*/ *Pteris* |
| HYMA05769 | A11 | 10.02.1977 | Northern Cape | 27.27S 23.26E | Kuruman | 41,568/13,388 | *Helianthus*/ *Helianthus* |
| HYMA05520 | A12 | 03.01.1970 | Transvaal (Gauteng) | 25.56S 28.13E | Olifantsfontein | 56,128/21,059 | *Helianthus*/ *Macrothamnium* |
| HYMA05608 | A13 | 11.12.1977 | Transvaal (North West) | 26.42S 27.05E | Potchefstroom (Tlokwe) | 3,624/119,717 | *Helianthus*/ *Astragalus* |
| HYMA05804/1 | A14 | 29.10.1985 | Transvaal (Gauteng) | 25.45S 28.12E | Pretoria at Gardens of Union Building | 27,679/26,117 | *Helianthus*/ *Pteris* |
| HYMA05804/2 | A15 | 29.10.1986 | Transvaal (Gauteng) | 25.45S 28.12E | Pretoria at Gardens of Union Building | 51,719/17,638 | *Helianthus*/ *Pteris* |
| HYMA05890 | A16 | 10.11.1981 | Natal (KwaZulu-Natal) | 28.55S 29.14E | Cathedral Peak Forestry Area | 61,489/103,851 | *Helianthus*/ *Pteris* |
| HYMA27298 | A17 | 11.01.1993 | Gauteng | 25.41S 28.18E | Roodeplaat Research Station | 122,274/4,913 | *Helianthus*/ *Pteris* |
| HYMA05911 | A18 | 24-25.02.1993 | Free State | 27.40S 25.45E | Sandveld Nature Reserve | 44,151/19,326 | *Helianthus*/ *Pleuropterus* |
| HYMA27299 | A19 | 24-25.02.1993 | Free State | 27.40S 25.45E | Sandveld Nature Reserve | 15,636/28,926 | *Helianthus*/ *Pteris* |
| HYMA27300 | A20 | 09.09.2007 | Northern Cape | 28.07S 17.00E | Richtersveld National Park at Hand of God | 38,647/23,219 | *Helianthus*/ *Pteris* |
| HYMA27301 | A21 | 19.01.2004 | Northern Cape | 27.13S 22.55E | 4 km West of Hotazel | 45,303/149,112 | *Helianthus*/ *Pteris* |
| HYMA27302 | A22 | 19.09.2005 | Western Cape | 32.12S 18.53E | South of Clanwilliam | 1,182/62,181 | *Helianthus*/ *Magnolia* |

^1^National Insect Collection’s (ARC, Pretoria) unique identifiers.

^2^South Africa has re-divided and renamed some of their provinces and towns. The original collection province and towns are given with the new names provided in brackets.
