# Supplementary material for: Plant–pollinator interactions over time: Pollen metabarcoding from bees in a historic collection
Source: Evol Appl. 2018 Nov 13;12(2):187–97. doi: 10.1111/eva.12707 (PMC6346658; doi:10.1111/eva.12707)
Supplement: Supplementary file 3 [file EVA-12-187-s003.docx]

# APPENDIX A: SCRIPT FOR CREATION OF SEQUENCE REFERENCE DATABASES FOR ITS1

A bioinformaticist colleague at the Biotechnology Platform, ARC, Mr. Jurgens de Bruin, developed The AVoGaDRo (AlternatiVe Genomics Dataset builder) script with my input. The script was designed to build sequence reference databases sourcing data from NCBI’s Genbank (Benson et al., 2015) that are compatible with three different classification software types, Greengenes (DeSantis et al., 2006), QIIME (Caporaso et al., 2010) and MiSeq Reporter as used in the Illumina 16S Metagenomic Sequence Workflow (Illumina Part #15042317 Rev. C, 2014). The script was mainly written in python, but utilized perl and sqlite as well. NCBI could be queried similarly to using the online resource by incorporating Biopython into the script. The final outputs were produced through various queries and data parsing commands.

The complete script is given below. Figure S1 precedes the script and aims to explain the different steps as a workflow, stating the important functions that perform each task. Script text is presented in a different font style and size.

|  |
| --- |
| **Figure S1: Workflow describing the different steps of the script developed by Mr. Jurgens de Bruin to create sequence reference databases by searching NCBI’s Genbank for sequence data and taxonomy information.** |

***AvoGaDRo: AlternatiVe Genomics Dataset BuildeR***

lg_pipeline_desc="""

Pipeline to assist in the creation of alternative MetaGenomics dataset to be used with MiSeqReport and Qiime.

This is full value pipline, thus if no dataset are available a NCBI query can be done and a full dataset will be created.

"""

ll_modules = ["Bio","csv",'re',"sys",'getopt','os',"stat","time","multiprocessing","operator","Bio.SeqRecord","mmap","linecache","fnmatch","sqlite3","argparse","subprocess"]

for ls_module in ll_modules:

try:

__import__(ls_module)

except ImportError:

print "Please install: %s" % ls_module

import sys,argparse

from Bio import Entrez

from BioSQL import BioSeqDatabase

from Bio import SeqIO

from Bio.Seq import Seq

import sqlite3

from Bio.SeqRecord import SeqRecord

from Bio.SeqIO.FastaIO import FastaWriter

from multiprocessing import Process, Pool, Manager

import subprocess

import string

import os

from Bio.SeqUtils.CheckSum import seguid

lg_parser = argparse.ArgumentParser(description=lg_pipeline_desc,add_help=True)

def create_sqlite_db(ls_sqlite_db):

if ls_sqlite_db.endswith(".db"):

ls_cmd_create = "sqlite3 %s/%s < ./biosql/sql/biosqldb-sqlite.sql" % (lg_outdir,ls_sqlite_db)

subprocess.call(ls_cmd_create, shell=True)

ls_load_ncbi_tax = "/biosql/scripts/load_ncbi_taxonomy.pl -dbname %s/%s --driver SQLite" % (lg_outdir,ls_sqlite_db)

subprocess.call(ls_load_ncbi_tax, shell=True)

else:

ls_cmd_create = "sqlite3 %s/%s.db < ./biosql/sql/biosqldb-sqlite.sql" % (lg_outdir,ls_sqlite_db)

subprocess.call(ls_cmd_create, shell=True)

ls_load_ncbi_tax = "./biosql/scripts/load_ncbi_taxonomy.pl -dbname %s/%s.db --driver SQLite"% (lg_outdir,ls_sqlite_db)

subprocess.call(ls_load_ncbi_tax, shell=True)

def create_sub_db(ls_sqlite_db,ls_sub_database,ls_genbank_file):

print ls_genbank_file

if ls_sqlite_db.endswith(".db"):

server = BioSeqDatabase.open_database(driver="sqlite3",db="%s/%s"%(lg_outdir,ls_sqlite_db))

else:

ls_sqlite_db += ".db"

server = BioSeqDatabase.open_database(driver="sqlite3",db="%s/%s"%(lg_outdir,ls_sqlite_db))

if ls_sub_database not in server.keys():

server.new_database(ls_sub_database)

else:

server.remove_database(ls_sub_database)

server.adaptor.commit()

server.new_database(ls_sub_database)

db = server[ls_sub_database]

lo_records_no_dup = remove_dup_seqs( SeqIO.parse(ls_genbank_file,"genbank") )

count = db.load(lo_records_no_dup)

server.commit()

def query_db_get_rank(ls_taxa,ls_sqlite_db):

ls_taxa = ''.join(ch for ch in ls_taxa if ch not in ["'"])

ls_sqlite_db = "%s/%s"%(lg_outdir,ls_sqlite_db)

if ls_sqlite_db.endswith(".db"):

conn = sqlite3.connect(ls_sqlite_db)

else:

ls_sqlite_db += ".db"

conn = sqlite3.connect(ls_sqlite_db)

cursor = conn.execute("SELECT node_rank from taxon where taxon_id = (SELECT taxon_id from taxon_name where name = '%s')"%ls_taxa)

try:

return cursor.fetchone()[0]

except:

return None

def create_header(ld_taxa,ls_taxon_header_template):

for key in ld_taxa:

#print key,ld_taxa[key]

#if key == 'superkingdom':

# ls_tmp = "sk__%s"%ld_taxa[key]

# ls_taxon_header_template = ls_taxon_header_template.replace("sk__",ls_tmp)

if key == "kingdom":

ls_tmp = "k__%s"%ld_taxa[key]

ls_taxon_header_template = ls_taxon_header_template.replace("k__",ls_tmp)

elif key == "phylum":

ls_tmp = "p__%s"%ld_taxa[key]

ls_taxon_header_template = ls_taxon_header_template.replace("p__",ls_tmp)

#elif key == "class":

# ls_tmp = "c__%s"%ld_taxa[key]

# ls_taxon_header_template = ls_taxon_header_template.replace("c__",ls_tmp)

#elif key == "subclass":

# ls_tmp = "sc__%s"%ld_taxa[key]

# ls_taxon_header_template = ls_taxon_header_template.replace("sc__",ls_tmp)

elif key == "order":

ls_tmp = "o__%s"%ld_taxa[key]

ls_taxon_header_template = ls_taxon_header_template.replace("o__",ls_tmp)

elif key == "family":

ls_tmp = "f__%s"%ld_taxa[key]

ls_taxon_header_template = ls_taxon_header_template.replace("f__",ls_tmp)

#elif key == "subfamily":

# ls_tmp = "sf__%s"%ld_taxa[key]

# ls_taxon_header_template = ls_taxon_header_template.replace("sf__",ls_tmp)

#elif key == "tribe":

# ls_tmp = "t__%s"%ld_taxa[key]

# ls_taxon_header_template = ls_taxon_header_template.replace("t__",ls_tmp)

#elif key == "subtribe":

# ls_tmp = "st__%s"%ld_taxa[key]

# ls_taxon_header_template = ls_taxon_header_template.replace("st__",ls_tmp)

elif key == "genus":

ls_tmp = "g__%s"%ld_taxa[key]

ls_taxon_header_template = ls_taxon_header_template.replace("g__",ls_tmp)

elif key == "species":

ls_tmp = "s__%s"%ld_taxa[key]

ls_taxon_header_template = ls_taxon_header_template.replace("s__",ls_tmp)

return ls_taxon_header_template

def write_fasta(ll_data,ls_genbank_name,ls_type):

ls_file = "%s/%s.%s.fasta"%( lg_outdir,lg_output_prefix,ls_type)

ls_outfile = open(ls_file,"wb")

lo_writer = FastaWriter(ls_outfile,wrap=0)

lo_writer.write_file(ll_data)

ls_outfile.close()

def finish(ll_greengenes,ll_qiime,ll_rdp):

ls_rdp = "%s/%s.RDP"%(lg_outdir,lg_output_prefix)

ls_outfile = open(ls_rdp,"wb")

ls_outfile.writelines("".join(ll_rdp))

ls_outfile.close()

for ls_type in ["GG","qiime"]:

if ls_type == "GG":

write_fasta(ll_greengenes,lg_output_prefix,ls_type)

elif ls_type == "qiime":

write_fasta(ll_qiime,lg_output_prefix,ls_type)

def create_datasets(ld_record,ll_greengenes,ll_qiime,ll_rdp_data,ls_sqlite_db):

print "Creating Data Sets"

print ld_record

ls_taxon_header = ""

ld_taxa = {}

for taxa in ld_record['taxonomy']:

ls_rank = query_db_get_rank(taxa,ls_sqlite_db)

ld_taxa[ls_rank] = taxa

print ld_taxa

#ls_taxon_header_template = "sk__;k__;p__;c__;sc__;o__;f__;sf__;t__;st__;g__;s__;"

ls_taxon_header_template = "k__;p__;o__;f__;g__;s__"

ls_taxon_header = create_header(ld_taxa,ls_taxon_header_template)

ls_header = "%s %s %s;" % (ld_record["id"],ls_taxon_header, ld_record['organism'])

lo_seq_greengenes = SeqRecord(Seq(ld_record["seq"]),id=ls_header,description="",name = ld_record["name"])

ll_greengenes.append(lo_seq_greengenes)

lo_seq_qiime = SeqRecord(Seq(ld_record["seq"]),id=ld_record["id"],description="",name = "")

ll_qiime.append(lo_seq_qiime)

ll_rdp_data.append("%s\t%s\n"%(ld_record["id"],ls_taxon_header.replace(" ","")))

#print "!!!!",len(ll_greengenes)

#print "!!!!",len(ll_qiime)

#print "!!!!",len(ll_rdp_data)

def query_NCBI(ls_qeury,ls_email):

#print ls_qeury

Entrez.email = ls_email

#lo_handle = Entrez.esearch(db="nucleotide",term=ls_qeury,usehistory="y",retmax=5)#testing purpose- remove later

lo_handle = Entrez.esearch(db="nucleotide",term=ls_qeury,usehistory="y")

lo_results = Entrez.read(lo_handle)

#print lo_results

webenv = lo_results["WebEnv"]

query_key = lo_results["QueryKey"]

#print lo_results["Count"]

batch_size = 1000

global lg_genbank_file

lg_genbank_file = "%s/%s.gb"%(lg_outdir,lg_output_prefix)

out_handle = open(lg_genbank_file, "w")

#for start in range(0,10,batch_size):#testing purpose- remove later

for start in range(0,int(lo_results["Count"]),batch_size):

end = min(int(lo_results["Count"]), start+batch_size)

print("Going to download record %i to %i" % (start+1, end))

fetch_handle = Entrez.efetch(db="nucleotide", rettype="gb", retmode="text",

retstart=start, retmax=batch_size,

webenv=webenv, query_key=query_key)

data = fetch_handle.read()

fetch_handle.close()

out_handle.write(data)

out_handle.close()

def remove_dup_seqs(records):

""""SeqRecord iterator to removing duplicate sequences."""

checksums = set()

try:

for record in records:

checksum = seguid(record.id)

if checksum in checksums:

#print "Ignoring %s" % record.id

continue

checksums.add(checksum)

yield record

except:

pass

def create_training():

ls_qiime_fasta = "%s/%s.qiime.fasta"%( lg_outdir,lg_output_prefix)

print ls_qiime_fasta,lg_genbank_file

ld_genbank_records = {}

for seq_record in SeqIO.parse(lg_genbank_file, "genbank"):

if seq_record.annotations["organism"] not in ld_genbank_records:

ld_genbank_records[seq_record.annotations["organism"]] = { "ID":[ seq_record.annotations["gi"] ], "Count" : 1}

else:

ld_genbank_records[seq_record.annotations["organism"]]["ID"].append(seq_record.annotations["gi"])

ld_genbank_records[seq_record.annotations["organism"]]["Count"] += 1

lo_no_duplicates_records = remove_dup_seqs(SeqIO.parse(ls_qiime_fasta, "fasta"))

count = SeqIO.write(lo_no_duplicates_records, "%s/%s.NoDup.fasta"%(lg_outdir,lg_output_prefix), "fasta")

print "Saved %i records" % count

ld_qiime = SeqIO.to_dict(SeqIO.parse("%s/%s.NoDup.fasta"%(lg_outdir,lg_output_prefix), 'fasta'))

ll_setB = []

ll_setA = []

ll_setT = []

for key in ld_genbank_records:

if ld_genbank_records[key]["Count"] == 1:

for ls_id in ld_genbank_records[key]["ID"]:

ll_setB.append(ld_qiime[ls_id])

else:

ll_setA.append(ld_qiime[ld_genbank_records[key]["ID"][0]])

for ls_id in ld_genbank_records[key]["ID"][1:]:

ll_setT.append(ld_qiime[ls_id])

print len(ll_setA)

print len(ll_setB)

print len(ll_setT)

print "Total", len(ll_setA) + len(ll_setB) + len(ll_setT)

SeqIO.write(ll_setA, "%s/%s_TrainingSetA.fa"%(lg_outdir,lg_output_prefix),"fasta")

SeqIO.write(ll_setT, "%s/%s_TrainingSetT.fa"%(lg_outdir,lg_output_prefix),"fasta")

SeqIO.write(ll_setB, "%s/%s_TrainingSetB.fa"%(lg_outdir,lg_output_prefix),"fasta")

def main(ls_sqlite_db,ls_gene_region, li_threads ):

if ls_sqlite_db.endswith(".db"):

server = BioSeqDatabase.open_database(driver="sqlite3",db="%s/%s"%(lg_outdir,ls_sqlite_db))

else:

ls_sqlite_db += ".db"

server = BioSeqDatabase.open_database(driver="sqlite3",db="%s/%s"%(lg_outdir,ls_sqlite_db))

db = server[ls_gene_region]

print "This database contains %i records" % len(db)

lo_pool = Pool(processes= li_threads)

lo_manager = Manager()

ll_greengenes = lo_manager.list()

ll_qiime = lo_manager.list()

ll_rdp_data = lo_manager.list()

for key,record in db.iteritems():

#record = db.lookup(gi=2921566)

ld_record = {"id":record.annotations['gi'],"taxonomy":record.annotations['taxonomy'],"organism":record.annotations['organism'],"seq":str(record.seq),"name":record.name}

#create_datasets(ld_record,ll_greengenes,ll_qiime,ll_rdp_data,ls_sqlite_db)

lo_pool.apply_async(create_datasets,args=(ld_record,ll_greengenes,ll_qiime,ll_rdp_data,ls_sqlite_db))

lo_pool.close()

lo_pool.join()

#print ll_greengenes

#print ll_qiime

#print ll_rdp_data

finish(ll_greengenes,ll_qiime,ll_rdp_data)

if lg_args.training:

create_training()

if __name__ == '__main__':

lg_parser.add_argument('-query', action="store", dest='ncbi_query', help='Search term to be used to qeury NCBI for specific gene region. Examples : txid33090[Organism:exp] internal transcribed spacer,txid33090[Organism:exp] rbcL ', type=str)

lg_parser.add_argument('-db', action="store", dest="sqlite_db", help="The name of BioSQL database", required=True)

lg_parser.add_argument('-email', action="store", dest="email", help="This is for NCBI")

lg_parser.add_argument('-db_create', action='store_true', default=False, dest="db_create", help="If the BioSQL database does not exist, specify True in order for a database to be create. If a database with the same name already exist it will be delete.")

lg_parser.add_argument('-update', action='store_true', default=False, dest="update", help="Update datasets.")

lg_parser.add_argument('-create', action='store_true', default=False, dest="create", help="Create Qiime and MiSeqReport")

lg_parser.add_argument('-gene_region', action="store", dest="gene_region", help="Specify the name of the gene regrion, example 16S,ITS.", required=True)

lg_parser.add_argument('-threads', action="store", dest="threads", help="Number of threads", required=True)

lg_parser.add_argument('-output_prefix', action="store", dest="output_prefix", help="Prefix for output files. Defaults to --gene_region")

lg_parser.add_argument('-output_dir', action="store", dest="output_dir", help="write all files to output directory",required=True)

lg_parser.add_argument('-training', action="store_true", default=False, dest="training", help="Create Training DataSets")

lg_parser.add_argument('-genbank_file', action="store", dest="genbank_file", help="If a genbank file exist , in which the data is present and NCBI query in not need please specify the file")

lg_parser.add_argument('--version', action='version', version='%(AVoGaDRo)s 1.0')

lg_args = lg_parser.parse_args()

print lg_args

global lg_output_prefix

lg_output_prefix = lg_args.output_prefix

global lg_outdir

ls_cwd = os.getcwd()

lg_outdir = "%s/%s"%(ls_cwd,lg_args.output_dir)

if os.path.exists(lg_args.output_dir) == False:

os.mkdir("%s/%s"%(ls_cwd,lg_args.output_dir))

# if lg_args.ncbi_query:

# if lg_args.email:

# query_NCBI(lg_args.ncbi_query,lg_args.email)

# else:

# print lg_args

# sys.exit(2)

if lg_args.db_create and lg_args.create:

create_sqlite_db(lg_args.sqlite_db)

print "Opened database successfully";

if lg_args.genbank_file and not lg_args.update:

print "Creating Sub-Database"

create_sub_db(lg_args.sqlite_db,lg_args.gene_region, lg_args.genbank_file)

print "Created Sub-Database"

main( lg_args.sqlite_db, lg_args.gene_region, int(lg_args.threads) )

elif lg_args.ncbi_query and not lg_args.update:

if lg_args.email:

query_NCBI(lg_args.ncbi_query,lg_args.email)

create_sub_db(lg_args.sqlite_db,lg_args.gene_region,lg_genbank_file)

main( lg_args.sqlite_db, lg_args.gene_region, int(lg_args.threads) )

else:

print lg_args

sys.exit(2)

***References***

Benson, D.A., Clark, K., Karsch-Mizrachi, I., Lipman, D.J., Ostell, J., Sayers, E.W., 2015. GenBank. Nucleic Acids Res 43, D30–D35. doi:10.1093/nar/gku1216

Caporaso, J.G., Kuczynski, J., Stombaugh, J., Bittinger, K., Bushman, F.D., Costello, E.K., Fierer, N., Peña, A.G., Goodrich, J.K., Gordon, J.I., Huttley, G.A., Kelley, S.T., Knights, D., Koenig, J.E., Ley, R.E., Lozupone, C.A., McDonald, D., Muegge, B.D., Pirrung, M., Reeder, J., Sevinsky, J.R., Turnbaugh, P.J., Walters, W.A., Widmann, J., Yatsunenko, T., Zaneveld, J., Knight, R., 2010. QIIME allows analysis of high-throughput community sequencing data. Nature Methods 7, 335–336. doi:10.1038/nmeth.f.303

DeSantis, T.Z., Hugenholtz, P., Larsen, N., Rojas, M., Brodie, E.L., Keller, K., Huber, T., Dalevi, D., Hu, P., Andersen, G.L., 2006. Greengenes, a chimera-checked 16S rRNA gene database and workbench compatible with ARB. Appl. Environ. Microbiol. 72, 5069–5072. doi:10.1128/AEM.03006-05
