# Supplementary material for: Plant–pollinator interactions over time: Pollen metabarcoding from bees in a historic collection
Source: Evol Appl. 2018 Nov 13;12(2):187–97. doi: 10.1111/eva.12707 (PMC6346658; doi:10.1111/eva.12707)
Supplement: Supplementary file 4 [file EVA-12-187-s004.docx]

# APPENDIX B: SCRIPT FOR TRIMMING

Quality and adapter trimming of reads were done using Trimmomatic 0.33 (Bolger *et al.* 2014) using the script provided below (substituting sequence names):

java -jar trimmomatic-0.33.jar PE -phred33 -trimlog ./trimlog.txt ITS1_S14_L001_R1_001.fastq.gz ./ITS1_S14_L001_R2_001.fastq.gz ./ITS1_R1_paired.fastq ./ITS1_R1_unpaired.fastq ./ITS1_R2_paired.fastq ./ITS1_R2_unpaired.fastq ILLUMINACLIP:NexteraPE-PE.fa:2:30:10 SLIDINGWINDOW:4:20 MINLEN:50

Thus all sequences with a length below 50 were discarded, and a quality score of 20 were applied for bases with a sliding window of 4 bases. Nextera adapters were trimmed from sequences in this same step.

***References***

Bolger AM, Lohse M, Usadel B (2014) Trimmomatic: a flexible trimmer for Illumina sequence data. *Bioinformatics*, **30**, btu170.
